# Maps of ticks (Acari: Argasidae, Ixodidae) for Austria and South Tyrol, Italy

**DOI:** 10.1007/s10493-022-00688-w

**Published:** 2022-01-20

**Authors:** Franz Rubel, Katharina Brugger

**Affiliations:** University of Veterinary Medicine Vienna, Veterinärplatz 1, 1210 Vienna, Austria

**Keywords:** Tick map, Species distribution, Georeferenced data, Cave fauna

## Abstract

A first compilation of georeferenced tick locations in Austria and South Tyrol, Italy, is presented here. This allows the tick fauna to be examined in the various climatic regions of the European Alps. The dataset comprises 424 tick locations of Austria and 48 tick locations of South Tyrol, which were digitized from literature and visualized in the form of geographical maps. The tick fauna of Austria includes two species of Argasidae in the genera *Argas* and *Carios* and 15 species of Ixodidae in the genera *Dermacentor*, *Haemaphysalis*, and *Ixodes*, altogether 17 tick species. In addition, two species of Ixodidae in the genera *Hyalomma* (each spring imported by migratory birds) and *Rhipicephalus* (occasionally imported by dogs returning from abroad with their owners) are included in the tick atlas. Of these, the georeferenced locations of 18 tick species are depicted in maps. The occurrence of the one remaining tick species, *Ixodes inopinatus*, is given at the level of the federal states. The first Austrian distribution map of the long-legged bat tick *Ixodes vespertilionis*, which was reported from 21 caves, deserves special mention. The most common and widespread tick species is *Ixodes ricinus*, with records in all nine federal states of Austria, followed by *Ixodes canisuga*, *Ixodes hexagonus*, and *I. vespertilionis* in six federal states each. *Haemaphysalis concinna* and *Dermacentor reticulatus* are only endemic in the eastern plains, while *Dermacentor marginatus* only occurs in the west, in the Tyrolean Alpine valleys. Eight tick species were reported from South Tyrol, Italy. There, the most frequently flagged tick from the vegetation is also *I. ricinus*, while *D. marginatus* and *Haemaphysalis punctata* are often collected from sheep. The locations are shown together with those from North and East Tyrol on a separate Tyrol map. The tick atlas in Austria and South Tyrol as well as the underlying digital dataset in the supplement contribute to the closing of data gaps in global distribution maps of ticks and improve the data basis for new species distribution models.

**Supplementary Information:**

The online version contains supplementary material available at 10.1007/s10493-022-00688-w.

## Introduction

Large parts of Austria are covered by the European Alps, which have a major impact on the climate and thus the distribution of tick species. According to the widely used Köppen–Geiger climate classification, the following climate zones can be distinguished in Austria: deciduous forest climate (abbreviation Cfb) at altitudes below 1100 m, mixed forest climate (Dfb, Cfc) at 1100–1400 m, boreal coniferous forest climate (Dfc) at 1400–2000 m, alpine tundra climate (ET) at 2000–3300 m, and alpine frost climate (EF) at the mountain tops (Rubel et al. 2017). For example, *Ixodes ricinus* has been mainly found in the warm temperate and boreal climate zones (Cfb, Cfc, Dfb, Dfc) up to 2000 m, but also in the alpine tundra climate (ET). In the latter, *I. ricinus* was collected from small mammals even up to 2500 m (Mahnert 1971). *Dermacentor reticulatus* is only widespread in the warm, temperate deciduous forest climate (Cfb) of eastern Austria (Rubel et al. 2016). *Dermacentor marginatus*, on the other hand, only occurs in the Alpine valleys of Tyrol (Thaler 2003) and South Tyrol, where the warm, temperate climate is characterized by hot summers (Cfa). The red sheep tick *Haemaphysalis punctata* is also found in South Tyrol (Simeoni et al. 2014). In order to quantitatively study the effects of climate change on the spread of ticks and tick-borne diseases, georeferenced tick locations are needed. Such datasets of the national tick faunas were compiled for Portugal (Santos-Silva et al. 2011), Great Britain (Jameson and Medlock 2011), Romania (Mihalca et al. 2012), Belgium (Obsomer et al. 2013), and Germany (Rubel et al. 2014, 2021). Nevertheless, there are still major gaps in the knowledge of the distribution of many tick species, including Austria and the neighbouring South Tyrol (autonomous province Alto Adige, Italy). Existing georeferenced datasets, such as those mapped in the scientific standard book *Ticks of Europe and North Africa* (Estrada-Peña et al. 2017), are therefore only a first step in describing the occurrence of tick species. For example, the map of the widespread hedgehog tick *Ixodes hexagonus* in that book (Sándor 2017) shows only one location in Austria and no location in South Tyrol. The tick atlas of Austria and South Tirol presented here is intended to provide not only printed maps but also digital data to help close gaps in the existing tick distribution maps.

Current studies on ticks and tick-borne diseases in Austria focus primarily on the initial description of newly emerging tick species (Duscher et al. 2018) and pathogens that have been detected in ticks (Blaschitz et al. 2008; Reiter et al. 2015; Walter et al. 2020). Recent research also focuses on forecasting next season’s *I. ricinus* density (Brugger et al. 2018) and the number of human tick-borne encephalitis cases (Rubel and Brugger 2020), also related to climate change (Rubel 2022). However, there is still no complete mapping of all tick species reported in Austria so far. The tick atlas presented here includes all 19 tick species found in Austria. There is also no checklist of ticks endemic in Austria published in the international literature, although Sixl and Nosek (1972) and Sixl (1972) described 15 tick species in their historical works written in German. Therefore, the atlas presented here can also be used as a checklist of the tick species occurring in Austria, which uses the current taxonomic status according to Petney et al. (2012) and Guglielmone et al. (2020). It is supplemented by a higher-resolution map in which tick species occurring in South Tyrol are shown. However, no information is given on the basic ecology as well as the medical and veterinary importance of the listed tick species. Hosts are also not described unless the ticks have been collected from them. Since all of the tick species described here also occur in neighbouring countries, reference is made to the annotated checklist of the ticks of Germany (Petney et al. 2012) and Hungary (Hornok et al. 2020). This information is therefore not repeated here, but reference is made to the relevant sources. An important additional information for assessing the reliability of the known tick locations in Austria is the description of the global distribution of each tick species. This has already been described in the atlas of ticks in Germany (Rubel et al. 2021) and has been adopted here for better readability. Therefore, the focus of this study is on the complete description of the known locations of all tick species in Austria and South Tyrol. To achieve this goal, tick locations described in the German- and Italian-language literature, which are difficult to access for the international scientific community, have been digitized.

## Data and methods

The data used here comprise 424 tick locations in Austria and 48 tick locations in South Tyrol, Italy. Tick locations were compiled from the literature by digitizing data or printed distribution maps, resulting in a data set of 472 georeferenced locations. The geographical coordinates of the new tick locations are provided in the supplement together with an indication of their accuracy and the sources. The coordinates are given in decimal degrees with a measure of accuracy divided into high (± 0.1 km), medium (± 1 km) and low (± 10 km) precision, identical to those previously used by Rubel et al. (2014, 2021).

**Fig. 1:**
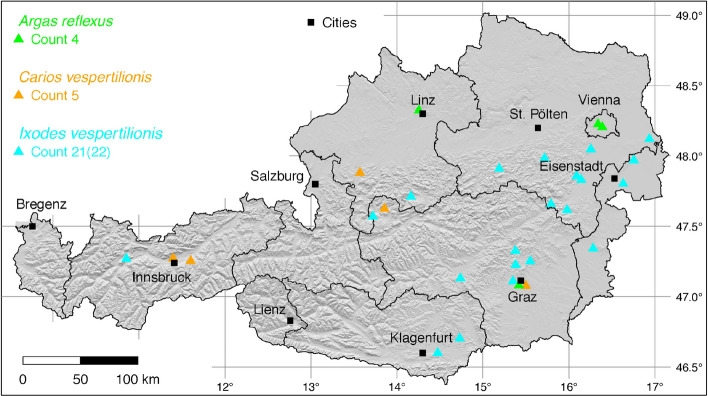
Recorded locations of *Argas reflexus*, *Carios vespertilionis*, and *Ixodes vespertilionis* in Austria

**Fig. 2:**
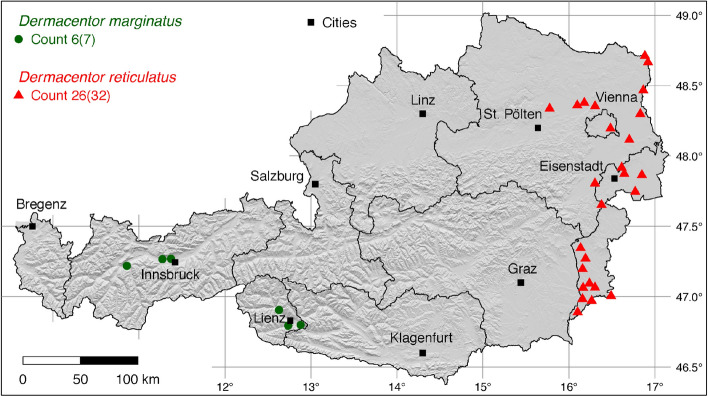
Recorded locations of *Dermacentor marginatus* and *Dermacentor reticulatus* in Austria

**Fig. 3:**
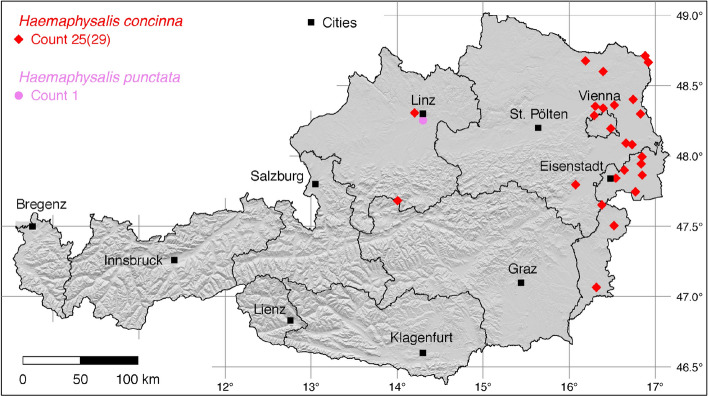
Recorded locations of *Haemaphysalis concinna* and *Haemaphysalis punctata* in Austria

**Fig. 4:**
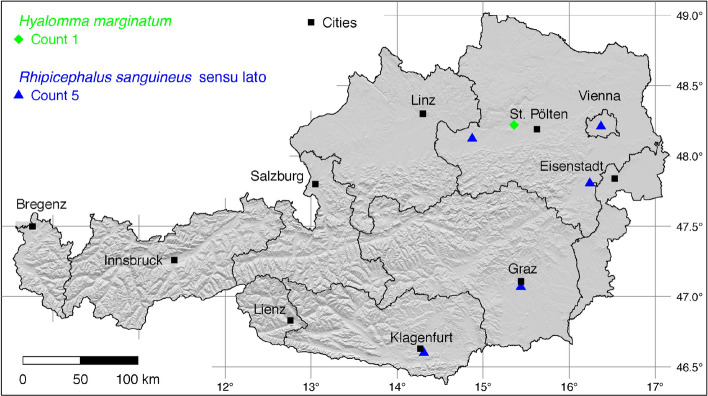
Recorded locations of *Hyalomma marginatum* and *Rhipicephalus sanguineus* in Austria. These species are not endemic in Austria, but are continuously introduced

**Fig. 5:**
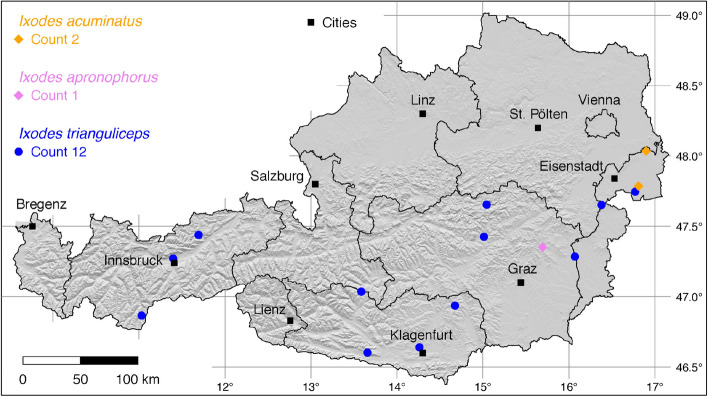
Recorded locations of *Ixodes acuminatus*, *Ixodes apronophorus*, and *Ixodes trianguliceps* in Austria

**Fig. 6:**
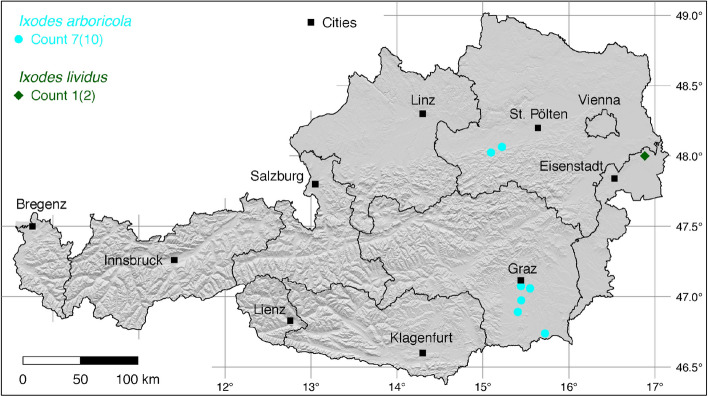
Recorded locations of *Ixodes arboricola* and *Ixodes lividus* in Austria

**Fig. 7:**
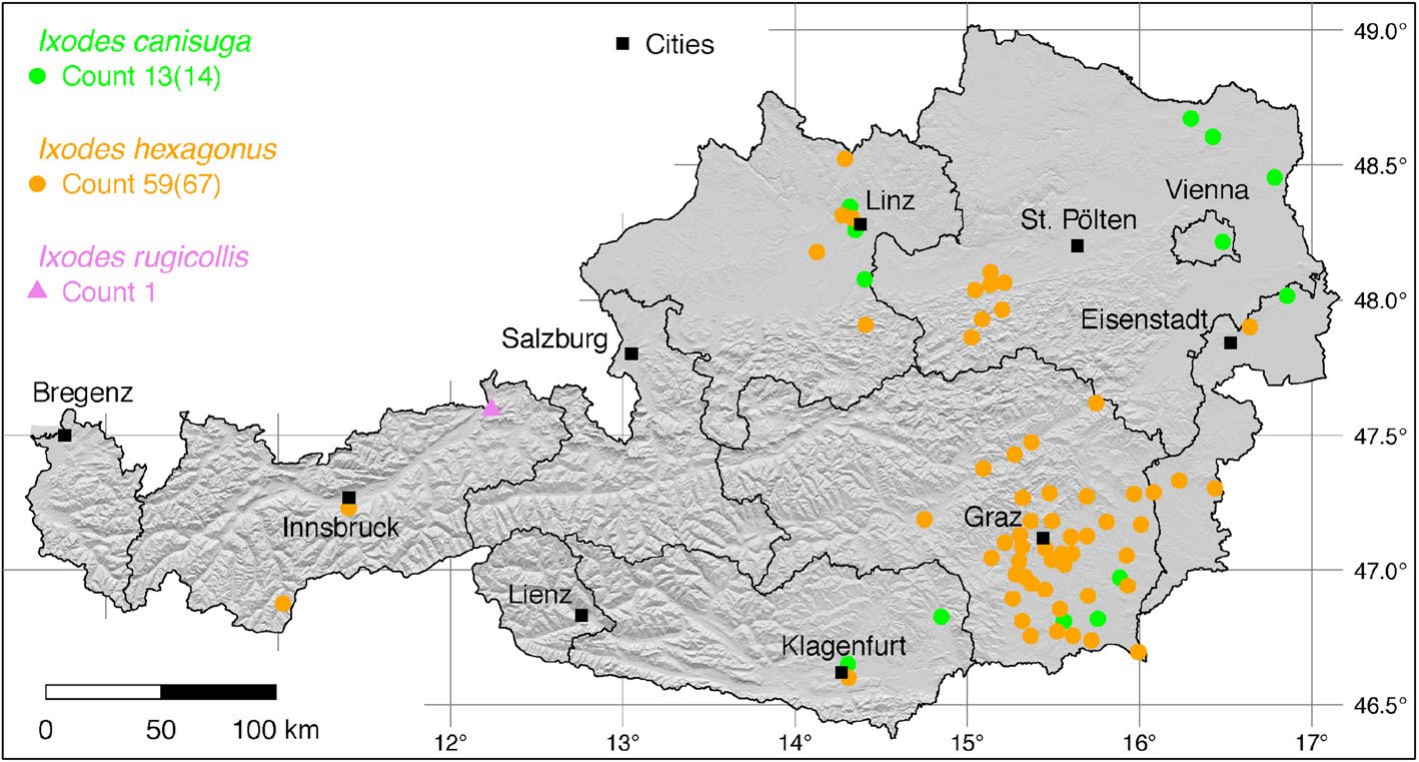
Recorded locations of *Ixodes canisuga*, *Ixodes hexagonus*, and *Ixodes rugicollis* in Austria

**Fig. 8:**
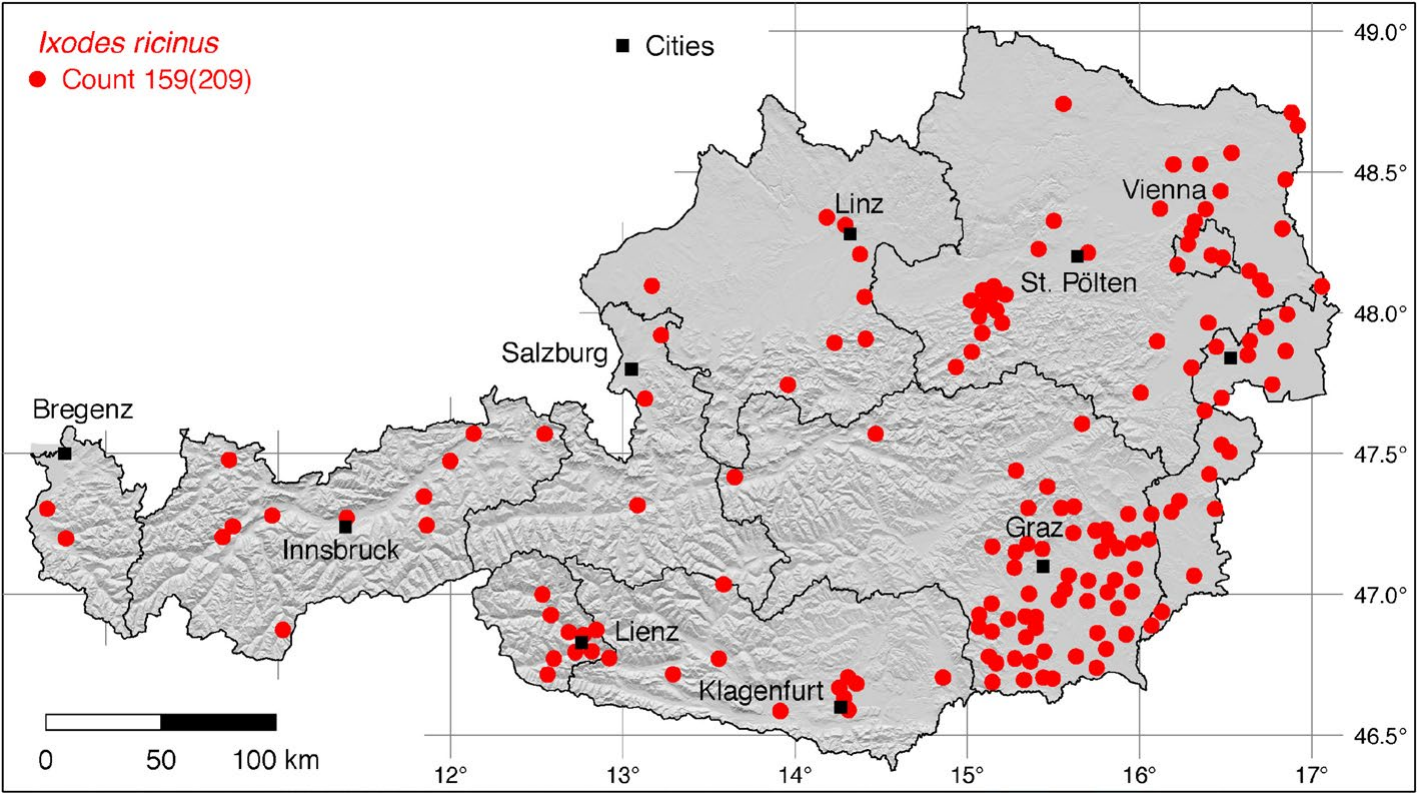
Recorded locations of the *Ixodes ricinus/inopinatus* species complex in Austria

**Fig. 9:**
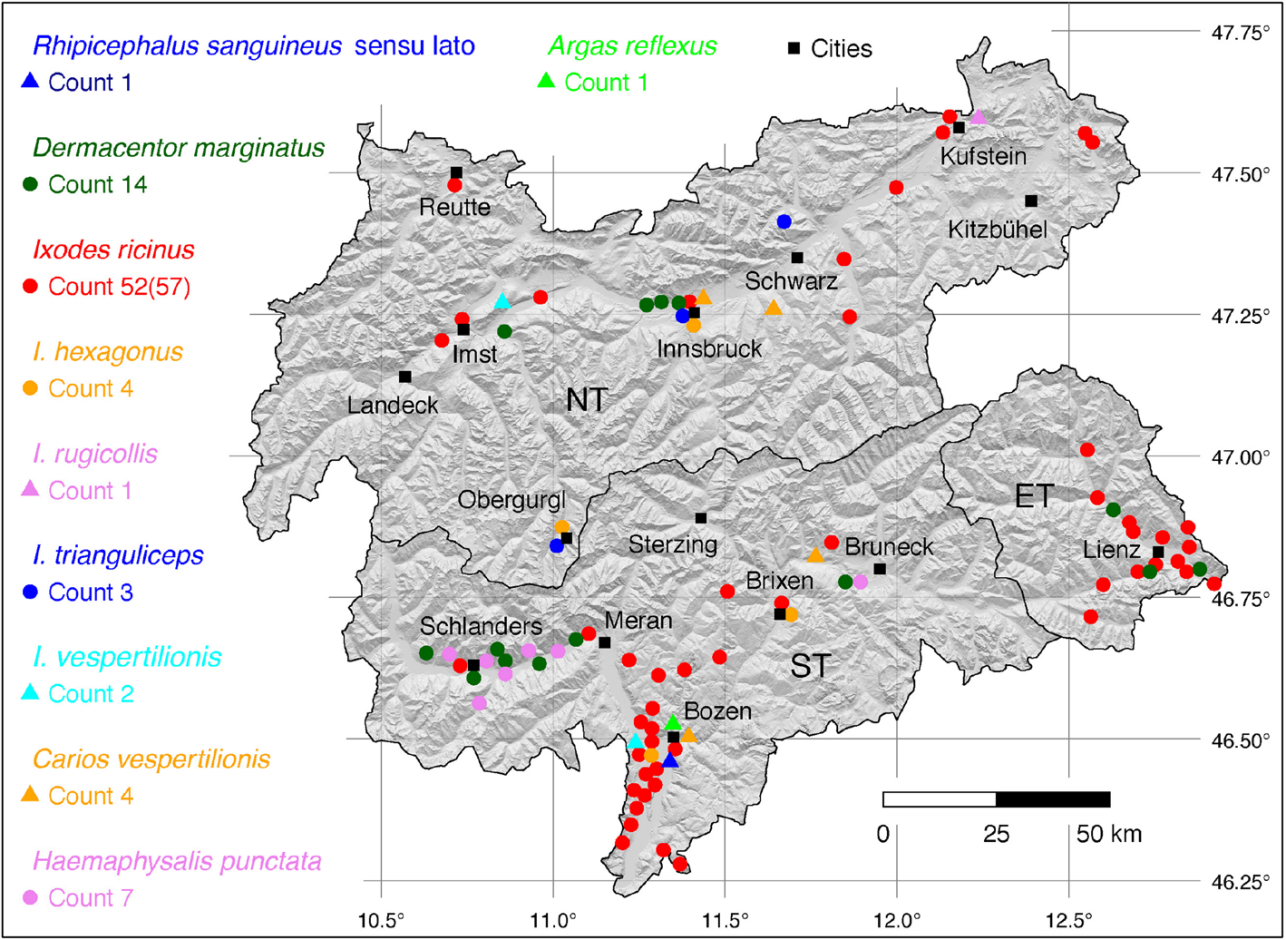
Recorded locations of ten tick species in Tyrol comprising North Tyrol and East Tyrol (NT and ET, federal state Tyrol, Austria) and South Tyrol (ST, autonomous province Alto Adige, Italy)

The tick locations are mapped using R, a language and environment for statistical computing (R Development Core Team 2019). However, they are not evenly distributed across the study area. For example, the federal state Styria is particularly well covered with data, since a research group has been dealing with the distribution of ticks and tick-borne diseases there (Sixl 1975d; Stünzner et al. 2006). This leads to an unusual clustering of known tick locations. In order to achieve a more realistic representation of the distribution of the individual tick species, these artificial clusters were reduced in a two-stage process. First, the tick locations of the two studies mentioned were reduced with the help of a random selection. For example, from the study by Sixl (1975d), only 68 of the more than 600 available *I. ricinus* locations were used. To further avoid local clustering and associated sampling biases in the dataset a spatial thinning algorithm was applied (Aiello-Lammens et al. 2015). The ’thin’ function in the spThin R package provided by Aiello-Lammens et al. (2019) uses a randomization approach and returns a dataset with the maximum number of locations for a given thinning distance, here 4–8 km for the maps of Austria (Figs. 1–8) and 2 km for the higher resolution map of Tyrol (Fig. 9). The maps for the individual tick species (Figs. 1–9) therefore not only show the number of tick locations mapped, but also the total number of available tick locations in brackets. A count of 143(186) for *I. ricinus* means that of the 186 tick locations available, only 143 were mapped.

## Results and discussion

The tick fauna of Austria includes two species of Argasidae in the genera *Argas* and *Carios* and 15 species of Ixodidae in the genera *Dermacentor*, *Haemaphysalis*, and *Ixodes*, altogether 17 tick species. In addition, two species of Ixodidae in the genera *Hyalomma* and *Rhipicephalus* were included in the atlas of ticks. Engorged nymphs of *Hyalomma marginatum* are imported by migratory birds each spring and unfed or feeding adults have been found for the first time in 2018 (Duscher et al. 2018). The tick *Rhipicephalus sanguineus* sensu lato has been occasionally imported by dogs returning with their owners from the Mediterranean or other countries with a subtropical or tropical climate (Prosl and Kutzer 1986). The tick fauna of South Tyrol includes six documented species (Canestri-Trotti and Fioravanti 1991; Khoury and Maroli 2004), one more than in North Tyrol (Mahnert 1971; Thaler 2003).

The outcome of this study is geographical maps that depict the occurrence of all tick species that have so far been reported in Austria and South Tyrol, Italy. The apparently widespread *I. inopinatus* (Estrada-Peña et al. 2014) is an exception. No map has been compiled for *I. inopinatus*, which is combined with *I. ricinus* to form the so-called *I. ricinus/inopinatus* complex here. This allows historical records of *I. ricinus* ticks to be mapped, which might occasionally include specimens that would now be identified as *I. inopinatus*. Although there is also quite a number of records of morphologically determined *I. inopinatus* in some parts of Austria (Vogelgesang et al. 2020), more work is needed to obtain a reliable identification of putative *I. inopinatus*.

Tick locations in Austria are shown in eight maps (Figs. 1–8). In addition, the locations throughout Tyrol are shown on a more detailed map. This map shows not only the ticks found in the Austrian federal state of Tyrol, but also those in the autonomous province South Tirol, Italy (Fig. 9) and thus gives a good overview of the occurrence of ticks both north and south of the main Alpine ridge.

Each tick species is presented below with a brief summary of its global distribution and the numbers of georeferenced locations in Austria and South Tyrol compiled for this study. If the ticks were collected from hosts, the involved species are also mentioned. Concerning any further details on the biology and ecology of the mentioned species, readers are referred to the excellent reviews by Petney et al. (2012) and Hornok et al. (2020).

### *Argas* (*Argas*) *reflexus* (Fabricius)

The pigeon tick, *A. reflexus* can be found from Portugal to the North Caucasus, in Europe up to 55$^{\circ }$ N (Dautel et al. 1991). *Argas reflexus* generally occurs in or close to the nests or resting places of their hosts (Dautel et al. 1999). The principal hosts of *A. reflexus* are domestic pigeons (*Columba livia domestica*) and rock pigeons (*Columba livia livia*), which are a characteristic component of many European towns. These pigeons live together with their parasites *A. reflexus* also in churches such as the Cathedral of Christ Church in Canterbury, the St. Mark’s Church in Venice or the Votive Church in Vienna (Schulze 1932). Because this tick can infest humans, especially when the natural host is not available for some years, it was an occasional cause of medical problems in the previous century. The appearance of the pigeon tick as a human parasite in Vienna was first published by Strouhal (1947). Records of buildings in Graz infested with *A. reflexus* in the 1970s have been documented by Sixl (1975c). In this study 1,600 pigeons and 45 pigeon-populated attics were examined. The occurrence of *A. reflexus* was classified as very common by Sixl (1975c). However, it can be assumed that the frequency of *A. reflexus* in Austrian cities has decreased in recent decades, as the number of city pigeons has been greatly reduced by official measures, and modern houses are not suitable for colonisation by pigeons. However, if there are problems with the occurrence of *A. reflexus*, professional pest controllers are called in. A scientific documentation of the cases has not been created. Therefore there are no descriptions of exact *A. reflexus* locations and the coordinates given here represent the occurrence in the respective city. Four locations have been documented: 1 (Schulze 1932), 1 (Strouhal 1947), 1 (Pfoser 1948), 1 (Sixl 1975c). A total of three known towns in which *A. reflexus* occurs is depicted in Fig. 1. In South Tyrol *A. reflexus* is known from Bozen (Khoury and Maroli 2004), as shown in Fig. 9.

### *Carios* (*Carios*) *vespertilionis* (Latreille)

The short-legged bat tick *C. vespertilionis* (also known as *Argas vespertilionis*) is widely distributed in the Old World from the Palaearctic to South Africa (Hoogstraal 1956). The distribution map recently presented by Sándor et al. (2021) shows the occurrence of *C. vespertilionis* up to 60$^{\circ }$ N latitude. The taxonomical status of this tick species like that of many other argasids is not certain. We follow the suggestion of Mans et al. (2021) to place it into the genus *Carios* as part of the Ornithodorinae. Main hosts are cave-dwelling, insectivorous bats, such as the common noctule (*Nyctalus noctula*) and the common pipistrelle (*Pipistrellus pipistrellus*) in Austria (Mahnert 1971). The following locations have been documented: 1 (Pfoser 1948), 1 (Mahnert 1971), 1 (Sixl et al. 1972), 2 (Sándor et al. 2021). A total of five known locations of the soft tick *C. vespertilionis* is mapped in Fig. 1. In South Tyrol, two *C. vespertilionis* locations were reported from the Bozen and Bruneck area (Sándor et al. 2021), which are shown together with two locations of North Tyrol in Fig. 9.

### *Dermacentor marginatus* (Sulzer)

The ornate sheep tick *D. marginatus* is mainly found in Mediterranean countries (Rubel et al. 2016) as well as in the Middle East and in countries of the former Soviet Union (Kulik and Vinokurova 1982). In China, its occurrence in the Uighur autonomous region of Xinjiang is confirmed (Teng 1982), although locations further east have also been described (Chen et al. 2010). It follows that the global distribution of *D. marginatus* extends from the Atlantic coast of Portugal to Western Siberia and Xinjiang, 9$^{\circ }$ W–92$^{\circ }$ E. In the north-south direction *D. marginatus* is distributed within the latitude belt of 33–58$^{\circ }$ N, while it occurs in Western and Central Europe only up to 51$^{\circ }$ N. In Austria, the tick is only found in Tyrol, where four locations have been documented in the Inn Valley (Thaler 2003) and three locations near Lienz (Kofler 2006). A total of six out of seven known locations is mapped in Fig. 2. In South Tyrol, the following locations have been documented: 3 (Canestri-Trotti and Fioravanti 1991), 4 (Simeoni et al. 2014). A total of 14 (four from North Tyrol, three from East Tyrol, and seven from South Tyrol) known locations in Tyrol, is mapped in Fig. 9.

### *Dermacentor reticulatus* (Fabricius)

The global distribution of the ornate dog tick *D. reticulatus*, also known as the marsh tick in Austria, extends from the Atlantic coast of Portugal to Western Siberia, 9$^{\circ }$ W–88$^{\circ }$ E, within the latitude range 34$^{\circ }$–60$^{\circ }$ N (Rubel et al. 2020). In Austria, *D. reticulatus* is particularly widespread and common in the eastern federal states of Vienna, Lower Austria, and Burgenland. There *D. reticulatus* occurs mainly along the Danube river (Weiler et al. 2017) and in the March-Thaya floodplains on the border with Slovakia and the Czech Republic (Hubálek et al. 1997). The following locations have been documented: 2 (Sixl 1975a), 2 (Hubálek et al. 1997), 1 (Dobler et al. 2008), 1 (Leschnik et al. 2012), 1 (Duscher et al. 2013), 8 (Duscher et al. 2013), 2 (Weiler et al. 2017), 10 (Hodžić et al. 2017), 1 (unpublished find by the authors, 2020), 4 (Wijnveld et al. 2021). A total of 26 out of 32 known locations is mapped in Fig. 2.

### *Haemaphysalis* (*Haemaphysalis*) *concinna* Koch

The global distribution of *Ha. concinna*, the relict tick, extends from the Spanish Atlantic coast to Kamchatka, 6$^{\circ }$ W–159$^{\circ }$ E (Rubel et al. 2018). In Europe, *Ha. concinna* occurs within the latitude belt of 40$^{\circ }$–56$^{\circ }$ N. It colonizes forest steppes and wet steppe habitats. In Austria, the distribution area of *Ha. concinna* corresponds very well to that of *D. reticulatus*, where both ticks are often reported together with *I. ricinus*. This is also known from Germany (Kahl et al. 1992). The following locations have been documented: 1 (Pfoser 1948), 14 (Sixl and Nosek 1971), 1 (Sixl and Nosek 1972), 2 (Hubálek et al. 2003), 1 (Blaschitz et al. 2008), 1 (Leschnik et al. 2012), 1 (Duscher et al. 2013), 1 (Fuehrer et al. 2013), 2 (Weiler et al. 2017), 2 (Vogelgesang et al. 2020), 4 (Wijnveld et al. 2021). A total of 25 out of 30 known locations is mapped in Fig. 3.

### *Haemaphysalis* (*Aboimisalis*) *punctata* Canestrini and Fanzago

The global distribution of *Ha. punctata*, also known as the red sheep tick, extends over the entire Mediterranean area of Europe and Northern Africa (Estrada-Peña et al. 2013) to Russia (Kolonin 2009) and China (Chen et al. 2010). The only report of *Ha. punctata* in Austria was documented in the historical paper by Pfoser (1948) in Linz, Upper Austria. The tick was found on the European polecat (*Mustela putorius*). It is likely to be a single finding, as the tick is not widespread north of the Alps, apart from occurrences 1,000 km away on Dutch (Hofmeester et al. 2016) and German (Rubel et al. 2021) North Sea coasts and islands. In the neighbouring areas of continental Germany and the Czech Republic there is no evidence of *Ha. punctata* and only individual findings were reported from Hungary (Hornok and Farkas 2009) and Slovakia (Bullova et al. 2009). The Austrian location is mapped in Fig. 3. In South Tyrol *Ha. punctata* occurs sympatric with *D. marginatus* in the Etsch Valley west of Meran and in the Bruneck area, both species frequently infesting sheep. The following locations have been documented: 3 (Canestri-Trotti and Fioravanti 1991), 4 (Simeoni et al. 2014). A total of seven known locations is mapped in Fig. 9.

### *Hyalomma* (*Euhyalomma*) *marginatum* Koch

The global distribution of *Hy. marginatum*, with the junior synonym *Hy. plumbeum* (Petney et al. 2012), extends over the Mediterranean area of Europe and Northern Africa to Western Siberia, 9$^{\circ }$ W–88$^{\circ }$ E (Kolonin 2009). Estrada-Peña et al. (2013) determined the northern distribution limit of *Hy. marginatum* south of the European Alps at about 45$^{\circ }$ N latitude. From there the Mediterranean *Hyalomma* tick is continuously introduced to northern countries by migratory birds although it has only been detected once in Austria. This adult tick was found on a horse in Melk, Lower Austria (Duscher et al. 2018). Numerous findings are known from neighbouring Germany, where *Hy. marginatum* was also found on horses or in horse stables (Chitimia-Dobler et al. 2019; Rubel et al. 2021). It is unclear at present whether or not *Hy. marginatum* has succeeded in establishing itself in parts of Austria. The same is true for *Hy. rufipes*, which was considered a subspecies of *Hy. marginatum* until it was recognized as a valid species. Currently there is no known location of *Hy. rufipes* in Austria (Hubálek et al. 2020). The only known *Hy. marginatum* location is depicted in Fig. 4.

### *Ixodes* (*Ixodes*) *acuminatus* Neumann

This tick species has a wide Palaearctic distribution, mainly in broad-leaved and mixed forests of the temperate climate zone (Guglielmone et al. 2014). In Austria, it was reported under the synonym *I. redikorzevi* in Parndorf and Apetlon, Burgenland (Sixl and Nosek 1972). There *I. acuminatus* infests the European ground squirrel (*Spermophilus citellus*), the European hamster (*Cricetus cricetus*) and the northern white-breasted hedgehog (*Erinaceus roumanicus*). The two known locations are depicted in Fig. 5.

### *Ixodes* (*Ixodes*) *apronophorus* Schulze

The tick is known from Eastern European forest steppes, the Carpathian mountain forests, Scandinavia, and the Russian taiga (Guglielmone et al. 2014). In Austria, two locations have been reported (Sixl and Nosek 1972). No information was given about the hosts, but it is known from the German site of Hagenburger Moor that *I. apronophorus* was found on small and medium-sized mammals such as the Eurasian water shrew (*Neomys fodiens*), the common vole (*Microtus arvalis*), the field vole (*Microtus agrestis*), the Eurasian harvest mouse (*Micromys minutus*), the striped field mouse (*Apodemus agrarius*), the yellow-necked mouse (*Apodemus flavicollis*), and the brown rat (*Rattus norvegicus*) (Walter 1980). In Austria, the tick was also found in moist, swampy habitats in Birkfeld, Styria. According to Sixl and Nosek (1972), it is certainly more widespread than is currently known. However, no geographical information is available for the second finding in Vorarlberg, which is why it could not be mapped here. The only known location is depicted in Fig. 5.

### *Ixodes* (*Pholeoixodes*) *arboricola* Schulze and Schlottke

The bird tick *I. arboricola* occurs in Northern Africa, Europe, Russia and China (Guglielmone et al. 2014). It is mainly found on cave breeders and it appears that it is widespread and moderately common in Austria. In southern and south-eastern Austria the tick was found in 17 of 385 examined tree cavities. In this study, Sixl and Nosek (1972) found the tick on birds such as the gray-headed woodpecker (*Picus canus*), the great spotted woodpecker (*Dendrocopos major*), the great tit (*Parus major*), the coal tit (*P. ater*), the willow tit (*Poecile montanus*), the Eurasian treecreeper (*Certhia familiaris*), the wood nuthatch (*Sitta europaea*), the collared flycatcher (*Ficedula albicollis*), and the European starling (*Sturnus vulgaris*). The following numbers of locations have been compiled for the present study: 6 (Sixl and Nosek 1972), 4 (Ressl 1995). A total of seven out of 10 known locations is depicted in Fig. 6.

### *Ixodes* (*Pholeoixodes*) *canisuga* Johnston

In Central Europe, *I. canisuga* frequently infests red foxes (*Vulpes vulpes*), which is why it is also known as the fox tick. The synonym *I. autumnalis* (Petney et al. 2012) was used by Pfoser (1948). According to Kolonin (2009), the tick is distributed from the Spanish Pyrenees to the east of China between 4.5$^{\circ }$W–144.0$^{\circ }$E and 32.5$^{\circ }$–58.5$^{\circ }$N. There are records from almost all European countries. In Asia, locations are known from Russia, Iran, Afghanistan, India (Kashmir), and China. The tick is native to all of Austria except in high alpine regions, although only a few georeferenced locations are documented. In addition to foxes, the tick is also found on dogs (Krebitz 1982; Leschnik et al. 2012). The following numbers of locations have been compiled for the present study: 4 Pfoser (1948), 1 (Sixl et al. 1971), 6 (Sixl and Nosek 1972), 1 (Krebitz 1982), 1 (Lassnig 1996), 1 (Leschnik et al. 2012). A total of 13 out of 14 known locations is depicted in Fig. 7.

### *Ixodes* (*Pholeoixodes*) *hexagonus* Leach

The occurrence of *I. hexagonus*, often referred to as the hedgehog tick, is limited to Europe (Kolonin 2009) and the neighbouring Turkey (Bursali et al. 2012). Documented locations range from Portugal, Northern Spain, and Great Britain to Central Europe, the Balkans, and Turkey. Accordingly, the distribution area ranges from 9.5$^{\circ }$ W to 41$^{\circ }$ E, within the latitude belt 37–59$^{\circ }$ N. In Austria, hedgehogs (*Erinaceus europaeus*), squirrels (*Sciurus vulgaris*), polecats (*Mustela putorius*), and otters (*Lutra lutra*) are documented hosts of *I. hexagonus* (Pfoser 1948). The most extensive study in Austria was carried out in Styria, where in the period 1968–1975 numerous ticks collected from dogs and cats by hunters and private individuals were sent in for species determination (Sixl 1975b). The tick was also found on 17 dogs in a veterinary practice in Klagenfurt, Carinthia, but without precise details of where the dogs were kept (Krebitz 1982). Dogs and cats are also often attacked by *I. hexagonus*. The following numbers of georeferenced locations have been compiled: 5 (Pfoser 1948), 8 (Mahnert 1971), 3 (Sixl 1971a), 36 (Sixl 1975b), 1 (Krebitz 1982), 1 (Sixl et al. 1989), 2 (Kaaserer et al. 1994), 8 (Ressl 1995), 3 (Lassnig 1996). A total of 59 out of 67 known locations is depicted in Fig. 7. In South Tyrol, the following locations have been documented: 1 (Canestri-Trotti and Fioravanti 1991), 1 (Simeoni et al. 2014). A total of two known locations is mapped in Fig. 9.

### *Ixodes* (*Ixodes*) *inopinatus* Estrada-Peña, Nava and Petney

This recently described tick species (Estrada-Peña et al. 2014) has been reported from Portugal, Spain, Germany, Austria, Romania, Morocco and Tunisia (Estrada-Peña 2017; Chitimia-Dobler et al. 2018; Younsi et al. 2020). Its exact global distribution has yet to be determined. Before *I. inopinatus* was described as a new species, it was identified as *I. ricinus*. Morphological identification of ticks collected in Vienna and Lower Austria demonstrated that 6.2% of all nymphal ticks and 1.6% of all adult ticks previously identified as *I. ricinus* may actually be *I. inopinatus* (Vogelgesang et al. 2020). This proportion of *I. inopinatus* is consistent with the data published for Germany (Chitimia-Dobler et al. 2018; Hauck et al. 2019). It can be assumed that *I. inopinatus* in sympatry with *I. ricinus* is endemic throughout Austria. No studies on *I. inopinatus* are known from South Tyrol. To continue to use historical locations and because the majority of recent studies in Europe have not yet differentiated between *I. ricinus* and *I. inopinatus*, the two species are combined herein and are referred to as the *I. ricinus/inopinatus* species complex. A separate map for *I. inopinatus* was therefore not compiled, although the species was found in the federal states Lower Austria and Vienna (Vogelgesang et al. 2020).

### *Ixodes* (*Pholeoixodes*) *lividus* Koch

The nest-dwelling bird parasite *I. lividus* typically infests sand martins (*Riparia riparia*) and house martins (*Delichon urbicum*). Its global distribution is between 9.5$^{\circ }$ W–145$^{\circ }$ E and 34$^{\circ }$–72$^{\circ }$ N (Kolonin 2009). In Austria, nymphs and larvae were found in Neusiedel and Parndorf (Burgenland) in nests of sand martins (Sixl 1971a; Sixl and Nosek 1972). There is no further research on the distribution of this tick species. One of the two neighbouring locations is depicted in Fig. 6.

### *Ixodes* (*Ixodes*) *ricinus* (L.)

The castor bean tick *I. ricinus* is widely distributed in the Western Palaearctic. It occurs from Portugal extending to the Volga river in Russia, and from the north of Finland to the Mediterranean countries including Northern Africa (Otranto et al. 2017). Due to climate change, its range has been expanding both northwards (Jaenson et al. 2012) and to higher mountain areas (Materna et al. 2008; Garcia-Vozmediano et al. 2020). In its distribution range, *I. ricinus* is the main vector of pathogens that cause tick-borne encephalitis and Lyme borreliosis, which is why it is the best-studied tick species in Europe. In addition, *I. ricinus* is also by far the most common tick species flagged from lower vegetation and collected from hosts. More than 600 such locations have been mapped in Styria (Sixl 1975d). Of these, however, only 68 randomly selected tick locations were taken over here to avoid artificial clustering on the map. The tick occurs throughout Austria, where it was collected from small mammals up to an altitude of 2,500 m (Mahnert 1971). The following numbers of georeferenced locations have been compiled: 7 (Pfoser 1948), 2 (Radda 1968), 1 (Radda et al. 1969), 2 (Mahnert 1971), 5 (Sixl 1971a), 1 (Sixl et al. 1972), 2 (Sixl 1975a), 68 (Sixl 1975d), 1 (Krebitz 1982), 13 (Radda et al. 1986), 1 (Radda 1988), 4 (Kaaserer et al. 1994), 17 (Ressl 1995), 3 (Lassnig 1996), 16 (Kofler 2002), 16 (Hubálek et al. 2003), 3 (Blaschitz et al. 2008), 1 (Dobler et al. 2008), 1 (Leschnik et al. 2012), 1 (Duscher et al. 2013), 1 (Fuehrer et al. 2013), 4 (Sonnleitner et al. 2015), 27 (Schötta et al. 2017), 2 (Weiler et al. 2017), 5 (Vogelgesang et al. 2020), 4 (Wijnveld et al. 2021), 1 (Kahl 2021). A total of 159 out of 209 digitized locations is depicted in Fig. 8. In South Tyrol, the following locations have been documented: 3 (Canestri-Trotti and Fioravanti 1991), 5 (Ciceroni et al. 1998), 19 (Simeoni et al. 2014). A total of 39 out of 41 (27 from North Tyrol and 14 from South Tyrol) known locations in Tyrol is mapped in Fig. 9.

### *Ixodes* (*Pholeoixodes*) *rugicollis* Schulze and Schlottke

Comparable to other related species such as *I. hexagonus* and *I. canisuga*, very little is known about the biology and distribution of this nidicolous tick, living in the nests of carnivores such as small mustelids (*Martes foina*, *M. martes*, *Mustela putorius*, *M. nivalis*), and red foxes (*Vulpes vulpes*) (Pfäffle et al. 2017). Only individual locations of *I. rugicollis* were reported from Central European countries. For example, Rubel et al. (2021) mapped five locations in Germany. Here, one georeferenced coordinate has been compiled from the Kaiser valley near Kufstein, Tyrol, where *I. rugicollis* was collected from a stone marten (*Martes foina*) by Visser et al. (2011). The known location is depicted in Fig. 7 and in the Tyrol map, Fig. 9.

### *Ixodes* (*Exopalpiger*) *trianguliceps* Birula

The shrew or vole tick *I. trianguliceps* is generally found in the nests and burrows of its small mammal hosts in the warm temperate and boreal climate zones of Eurasia. The distribution area between 9$^{\circ }$ W–88$^{\circ }$ E and 43$^{\circ }$–70$^{\circ }$ N extends from Northern Spain to Western Siberia, but *I. trianguliceps* does not occur in the Mediterranean area (Kolonin 2009). In the Tyrolean Alps, the tick was found on the yellow-necked mouse (*Apodemus flavicollis*), bank vole (*Myodes glareolus*), shrews (*Sorex alpinus, S. araneus, S. minutus*), field vole (*Microtus agrestis*), and snow vole (*Chionomys nivalis*) at altitudes of 1,000–2,300 m (Mahnert 1971). It can be assumed that the tick is widespread throughout Austria, although no recent data are available. The following numbers of locations were digitized: 3 (Mahnert 1971), 5 (Sixl et al. 1971), 2 (Sixl and Nosek 1972), 2 (Sixl 1975a). A total of 12 known locations is depicted in Fig. 5.

### *Ixodes* (*Eschatocephalus*) *vespertilionis* Koch

The long-legged bat tick *I. vespertilionis* (syn. *Eschatocephalus gracilipes*) is widespread in Central and Southern Europe and reported from Africa, Middle East, Southeast Asia, Pacific islands, China, and Japan (Ševčik et al. 2010). However, there is no location in Austria on the only previously available distribution map of *I. vespertilionis* (Hornok 2017), although numerous locations in the neighbouring countries of Italy and Slovenia suggest an occurrence in Austria. In fact, the first descriptions of this cave-dwelling bat tick in Austria were made in the 19th century under the synonym *Sarconissus flavipes* Koch, where it was found on lesser horseshoe bats (*Rhinolophus hipposideros*) in the Hermannshöhle (Kolenati 1857). Approximately a hundred years later, several locations were already known from bat caves and grottos in Lower Austria, Burgenland and Styria (Vornatscher 1960). In 1975 the catalogue of the extant cave animals of Austria (Strouhal and Vornatscher 1975) and at the same time a description on the land fauna of the Lurgrotte (Neuherz 1975) were published, in which all known locations were listed. The long-legged bat tick was reported from the following caves: Lurgrotte near Semriach, Drachenhöhle near Mixnitz, Kurathöhle near Grafenstein, Griffener Tropfsteinhöhle, Knappenlöcher on the Tschirgant mountain near Magerbach, Koppenbrüllerhöhle near Obertraun, Kreidelucke near Hinterstoder, Dreidärrischenhöhle near Mödling, Einhornhöhle at the Hohe Wand, Falkensteinhöhle near Breitenstein at the Semmering, Güntherhöhle near Hundsheim, Hermannshöhle near Kirchberg am Wechsel, Taubenloch on the Ötscher mountain, Türkenloch near Kleinzell, Tropfsteinhöhle Katerloch near Weiz, Graselhöhle near Weiz, Schafferloch near Weißkirchen, Fledermauskluft near Sankt Margarethen, Bärenhöhle near Winden, Eisensteinhöhle near Fischau, Stiller-Graben-Stollen near Goberling. There *I. vespertilionis* parasitizes a variety of bat species such as the greater horseshoe bat (*Rhinolophus ferrumequinum*), the lesser horseshoe bat (*R. hipposideros*), the greater mouse-eared bat (*Myotis myotis*), the lesser mouse-eared bat (*Myotis blythii*), or the Natterer’s bat (*Myotis nattereri*). The following numbers of locations that have not yet been shown on any tick map have been digitized: 1 (Vornatscher 1960), 1 (Sixl et al. 1972), 5 (Neuherz 1975), 14 (Strouhal and Vornatscher 1975), 1 (Pavuza et al. 2018). It can be assumed that *I. vespertilionis* is widespread throughout Austria and there are also numerous other, undocumented occurrences in bat caves. A total of 21 out of 22 digitized locations is depicted in Fig. 1. In South Tyrol, a cave locations near Bozen was digitized from Hornok (2017). A total of two (one from North Tyrol and one from South Tyrol) known locations in Tyrol is mapped in Fig. 9.

### *Rhipicephalus sanguineus* sensu lato

What was originally called the brown dog tick *R. sanguineus* is a complex of closely related species called *Rhipicephalus sanguineus* sensu lato (Nava et al. 2015, 2018). It is the most common tick found on dogs (Dantas-Torres and Otranto 2017) and is reported worldwide within the latitude belt 42$^{\circ }$ S–46$^{\circ }$ N (Kolonin 2009). In the Mediterranean area, georeferenced locations of *R. sanguineus* s.l. have been mapped by Estrada-Peña et al. (2013). Accordingly, the natural northern distribution limit of *R. sanguineus* s.l. is currently south of the Alps at 46$^{\circ }$ N latitude. Locations north of the Alps have also been known for a long time, e.g., in Austria (Hinaidy and Tschepper 1979) and former Czechoslovakia (Černý 1985). However, they can be traced back to introductions on dogs by travelers returning from abroad. Afterwards a massive tick outbreak was often observed in the houses or apartments of the dog owners, as documented by Prosl and Kutzer (1986). Another study reported *R. sanguineus* s.l. on eight dogs in a veterinary practice in Klagenfurt, Carinthia, but without exact information on the locations (Krebitz 1982). Often only the treatment of the infections transmitted by *R. sanguineus* indicated an infestation with the tick. For example, Schwendenwein (1998) describes clinical manifestations, diagnosis and treatment of *Babesia canis* in dogs with massive tick infestations after returning from Turkey and Libya. Dogs infested with *Rhipicephalus* spp. that were infected with *Ehrlichia canis* in an Austrian animal welfare station were mentioned by Leschnik et al. (2008). The following numbers of locations were digitized from three historical studies: 1 (Sixl 1972), 1 (Krebitz 1982), 3 (Prosl and Kutzer 1986). A recent occurrence from Bratislava (Slovak Republic), approximately 50 km east of Vienna, in 2021 indicates that *R. sanguineus* s.l. is still being introduced to Central European cities (Didyk et al. 2021). It is noteworthy that the mentioned findings do not reflect geographic distribution of an established population but are the results of sporadic findings. As a rule, such outbreaks have been eliminated by pest controllers. Despite the likely more frequent occurrence of the tick, only five georeferenced locations could be mapped in Fig. 4. In South Tyrol *R. sanguineus* s.l. is known from Bozen (Canestri-Trotti and Fioravanti 1991), as shown in Fig. 9.

## Conclusions

A first compilation of tick maps, referred to as atlas of ticks in Austria and South Tyrol, Italy, has been presented here. Despite this extensive collection of georeferenced tick locations, there are still considerable gaps in our knowledge of the occurrence of several tick species in Austria and South Tyrol. For example, there is no evidence of the occurrence of *Ixodes frontalis*, although this tick species has been found in all neighbouring countries (Pfäffle et al. 2017). Only a decade after the first German record of *I. frontalis* Schorn et al. 2011, the current distribution map shows records of *I. frontalis* throughout Germany (Rubel et al. 2021). It can therefore be assumed with a high degree of probability that *I. frontalis* also occurs in Austria. Another question is how abundant the pigeon tick *A. reflexus* still is in Austria. That there are no recent reports can also mean that the abundance of the pigeon tick is much lower now than in the 20th century. In addition, there are no recent publications on the occurrence of the brown dog tick *R. sanguineus* s.l., although, due to the great increase in travel activity in recent decades, it can be assumed that this Mediterranean tick species is still imported frequently to Austria and South Tyrol.

At this point it must be mentioned that rarely detected tick species whose distribution area is definitely not in Central Europe are not taken into account here. This includes the single records of *Rhipicephalus turanicus* on a dog in Kitzbühel, Tyrol (Sixl 1972) and *Hyalomma aegyptium* on a tortoise imported from the Balkans, the former Yugoslavia (Sixl 1971b). *Hyalomma aegyptium* is the dominant tick on tortoises of the genus *Testudo* in Balkan countries (Široký et al. 2006) and in Northern Africa (Gemel and Hörweg 2011). Due to the strict species protection regulations, hardly any tortoises and with them *Hy. aegyptium* are imported into Austria today.

Finally, the results of the tick mapping for the individual federal states of Austria are summarized. Table 1 shows that only *I. ricinus* has been documented in all nine federal states. The occurrence of *I. inopinatus* reported from two federal states has been marked with a circle because it is not shown in a separate map. *Ixodes ricinus* described in older studies would have included about 1–7% of *I. inopinatus* (Vogelgesang et al. 2020), which might be distributed throughout Austria. Further occurrences of ticks marked with a circle, for which no coordinates are provided and which are therefore not shown on a map, concern *I. apronophorus* in Vorarlberg (Sixl and Nosek 1972). The common tick species *I. hexagonus* and *I. canisuga* were reported in six federal states. Together with *A. reflexus*, which was reported only in three federal states, they may also occur throughout Austria. However, this has not yet been documented. The ornate tick *D. reticulatus* is exclusively distributed in the east of Austria and after *I. ricinus* the second most common tick species flagged from the vegetation. In eastern Austria, *Ha. concinna* is also frequently flagged from vegetation, but also found on wild ruminants such as roe deer (*Capreolus capreolus*) and red deer (*Cervus elaphus*) (Kutzer and Hinaidy 1969). At the other end of the frequency distribution there are six tick species that have only been detected in one federal state.

**Table 1:**
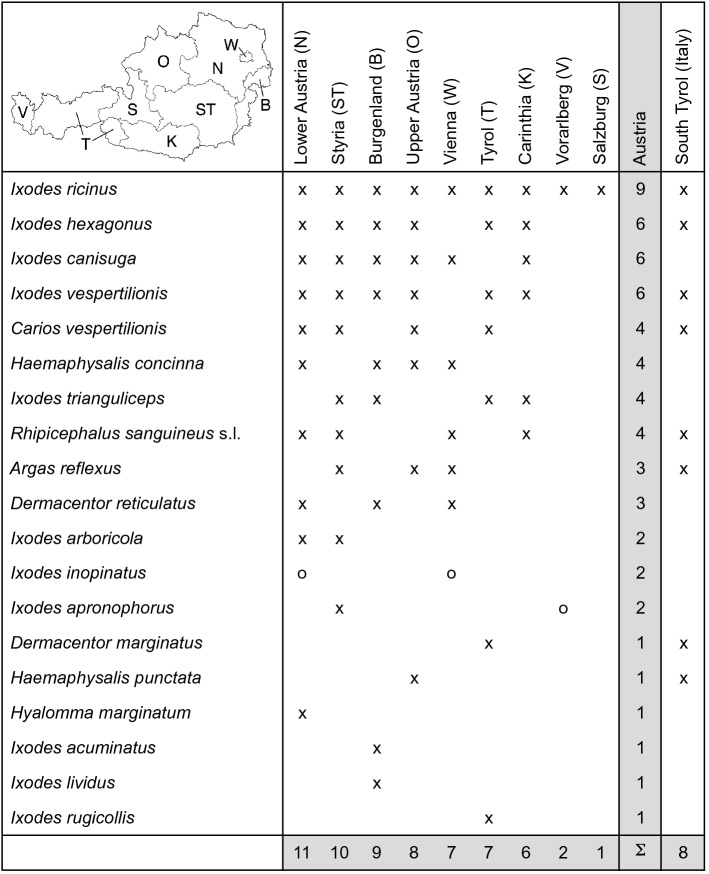
Occurrence of 19 tick species (Acari: Argasidae, Ixodidae) in the nine Austrian federal states and eight tick species in South Tyrol, Italy: x) georeferenced locations of this study and o) documented in the scientific literature

The total number of tick species per federal state as well as the total number of Austrian federal states in which a tick species occurs are highlighted in gray

Table 1 shows how many tick species have been found in each federal state, according to which Lower Austria leads with 11 documented tick species, followed by Styria with ten, Burgenland with nine, and Upper Austria with eight documented tick species. All these federal states are located in the eastern part of Austria. These statistics show that knowledge about the tick fauna is significantly less in some western federal states. In the absence of field studies, only the occurrence of the most common tick species *I. ricinus* was documented in Salzburg. Eight tick species were mapped in South Tyrol.

## Supplementary Information

Below is the link to the electronic supplementary material.

Supplementary file 1 (XLSX 49 kb)
